# Influence Mechanism of Vermicompost with Different Maturity on Atrazine Catabolism and Bacterial Community

**DOI:** 10.3390/toxics13010030

**Published:** 2025-01-01

**Authors:** Luwen Zhang, Lixin Xu, Zunhao Zhang, Jiaolin Li, Limeng Ren, Zhichen Liu, Yan Zhang, Yuxiang Chen

**Affiliations:** 1 College of Biological and Agricultural Engineering, Jilin University, Changchun 130022, China; luwen20@mails.jlu.edu.cn (L.Z.); liermu@foxmail.com (J.L.); renlm22@mails.jlu.edu.cn (L.R.); liuzhichen1996@163.com (Z.L.); 2 College of Life Sciences, Jilin University, Changchun 130012, China; xlx626@jlu.edu.cn; 3 The Electron Microscopy Center, Jilin University, Changchun 130000, China; zhangzunhao@jlu.edu.cn; 4 Costal Research and Extension Center, Mississippi State University, Pascagoula, MS 39567, USA; yzhang@fsnhp.msstate.edu

**Keywords:** vermicompost, atrazine, microbial communities, metabolic pathways, co-occurrence networks

## Abstract

Atrazine causes serious contamination of agricultural soils and groundwater. This study investigated the influence mechanism of sterilized soil (CKs), unsterilized soil (CKn), sterilized soil amended with 45 (SsV1), 60 (SsV2), 75 (SsV3) days of vermicompost (the maturity days of vermicompost), and unsterilized soil amended with 45 (SnV1), 60 (SnV2), 75 (SnV3) days of vermicompost on atrazine catabolism. The atrazine degradation experiment lasted for 40 days. The results showed that the atrazine degradation rates for CKs, CKn, SsV1, SsV2, SsV3, SnV1, SnV2, and SnV3 were 24%, 56.9%, 62.8%, 66.1%, 65.9%, 87.5%, 92.9%, and 92.3%, respectively. Indigenous microorganisms capable of degrading atrazine were present in unsterilized soil, and the addition of vermicompost enhanced atrazine degradation. The humic acid content of SnV2 was the highest, at 4.11 g/kg, which was 71.97% higher than that of CKn. The addition of the vermicompost enhanced the production of hydroxyatrazine, deethylatrazine, and deisopropylatrazine. Vermicompost increased the abundance of atrazine-degrading bacteria (*Mycobacterium*, *Devosia*, etc.), and introduced new atrazine-degrading bacteria (*Mesorhizobium*, *Demequina*). The above results showed that the best degradation of atrazine was achieved with 60 days of vermicompost addition. This study provides a new, efficient, economical, and environmentally friendly strategy for the remediation of atrazine-contaminated soil.

## 1. Introduction

Atrazine (2-chloro-4-ethylamino-6-isopropylamino-1,3,5-triazine) is a widely used triazine herbicide that is used to control annual grasses and broadleaf weeds. As one of the most widely used pesticides in the world, the global annual use of atrazine is about 70,000–90,000 tons [1]. In the United States, the annual use of atrazine exceeds 35,000 tons [2]. In India, about 340 tons of atrazine are consumed annually [2]. In Japan, 1.75 × 10^3^ tons of atrazine are used annually [3]. In China, the annual consumption of atrazine (2019) is close to 13,000 tons and then expanded by 20% per year [4]. Atrazine is ubiquitous in agricultural soils and aquatic systems due to its high persistence, ease of transport, and long-lasting presence in soil and water across decades [5,6]. Previous studies have reported atrazine concentrations reaching 175 μg/L in natural waters in Nebraska, USA [6]. The highest concentrations of atrazine in greenhouses and open soils in China have been recorded at 137 and 134 ng/g, respectively [5]. Widespread use of atrazine not only causes long-term residual contamination of agricultural soils but also poses a potential threat to subsequent crops, ecosystems, and human health [7,8,9]. Atrazine produced phytotoxicity to alfalfa at a concentration of 0.1 mg/L and reduced its chlorophyll content and membrane permeability [10]. Atrazine seriously inhibited the growth of rice roots and buds and reduced the biomass of roots and buds by 48.9% to 79.8% when the concentration of atrazine reached 0.4 mg/L^−1^ [11]. Hayes et al. showed that adult male frogs gradually lost their male reproductive ability and gradually became feminized after exposure to atrazine; even if the concentration of atrazine in the aqueous environment was as low as 0.1 μg/L it would have an adverse effect on the reproductive system of male frogs [7]. In addition, the immune system and endocrine system of human beings are threatened after long-term exposure to atrazine. Moreover, the results of the survey showed that the incidence of prostate cancer in men with long-term exposure to atrazine was 4.5 times higher than that of normal men, and the incidence of ovarian cancer and breast cancer in women with long-term exposure to atrazine was also high [8]. Therefore, it is very important for agricultural and environmental safety to research methods for the efficient and swift removal of atrazine.

Some of the previously reported methods for remediation of atrazine-contaminated soil include physical adsorption, chemical oxidation, washing, and microbial decomposition and mineralization [12,13,14,15]. Bioremediation is the most environmentally friendly method to removal atrazine, as evidenced by previous research reports. However, the indigenous microorganisms in the soil that can mineralize atrazine grow slowly, have low abundance, poor catabolic activity, and relatively low degradation efficiency [15]. In view of this, many researchers have enhanced microbial metabolic activity through bioaugmentation to effectively remove atrazine, which is difficult to degrade in the environment [16,17,18]. For example, the degradation of atrazine in soil can be accelerated by inoculating strains carrying atrazine-degrading genes (*atz* and *trz* families) into soil. The strains such as *Arthrobacter* sp. C2 and *Bacillus* subtilis Strain HB-6 converted atrazine to cyanuric acid by their own *atzA*/*trzN*, *atzB*, and *atzC* gene products [19,20]. However, the inoculated strains often have poor environmental adaptability and low survival rate, which resulted the degradation efficiency of atrazine easily affected [21]. Moreover, the biodegradation of atrazine in the soil is often limited by many environmental variables, such as nutrient levels, pH, and the C:N:P ratio [22]. In view of the above reasons, bioaugmentation using organic fertilizers, such as manure, straw, and compost, is a promising approach to improve the bioremediation performance of atrazine pollution [22,23].

Vermicompost prepared with maize stover and cattle dung serves as an efficient and stable organic fertilizer that has a higher nutrient content essential for plants and contains a more diverse array of agricultural and aquaculture probiotics than other organic fertilizers [24]. It is now considered to be both an effective and environmentally friendly organic fertilizer for the removal of soil organic pollutants [17]. Previous studies have shown that the use of vermicompost can effectively remove organic pollutants, such as total petroleum hydrocarbons (TPHs), tetracyclines, and polycyclic aromatic hydrocarbons (PAHs) [25,26]. Moreover, vermicompost plays an important role in improving soil physicochemical properties, increasing soil basal respiration, stimulating native microbial activity, and accelerating the degradation of organic pollutants [26,27]. Lin et al. (2016) demonstrated that vermicompost activated the degradation of pentachlorophenol (PCP) by increasing the available substrate of native microorganisms [27]. Zhang et al. (2023) used vermicompost to increase the content of organic fractions, such as organic carbon and humus, which increased the adsorption of atrazine and reduced the migration of atrazine in the environment [28]. At the same time, there are also a variety of earthworm intestinal microorganisms in the vermicompost, among which *Cupriavidus*, *Pseudomonas*, and *Flavobacterium* are related to atrazine mineralization [29]. Therefore, vermicompost can improve the soil microenvironment by increasing nutrients and organic matter required by plants [30] and promote the degradation of pollutants by introducing intestinal microorganisms. To date, the degradation mechanism of atrazine by different organic fertilizers has been widely studied, but there is relatively little research has been conducted on vermicompost as a bio-organic soil amendment, and the mechanism of vermicompost on atrazine degradation is still unclear. Specifically, the research information on the bioremediation of atrazine-contaminated soil using vermicompost with different maturity is limited. Vermicompost with different maturity has different nutrients, physicochemical properties, and microbial community structure, which can influence the degradation efficiency of atrazine in bioremediation. Therefore, in this study, maize stover and cattle dung were used as raw materials, and the two materials were mixed in equal proportions to prepare the vermicompost. Vermicompost was used as a soil bio-organic amendment by the vermicompost with a 45-, 60-, and 75-day maturity, respectively. The role of vermicompost with different maturity on the biodegradation of atrazine in soil was investigated. The effects of vermicompost with different maturity on the degradation performance of atrazine, soil physicochemical properties, and bacterial community structure were studied. The response of microbial community structure and function to atrazine degradation was analyzed in conjunction with PICRUSt2. The objectives of this study were (1) to analyze the degradation rate of atrazine and the effects of different maturity of vermicompost on atrazine metabolic pathways; (2) to explore the changes in soil microbial community structure after vermicompost treatment with different maturity; and (3) to investigate the correlation between atrazine metabolic pathways, soil microorganisms, and soil physicochemical properties in soil amended with vermicompost. The results of this study will elucidate the mechanism of vermicompost with different maturity on the biodegradation of atrazine in soil so as to make scientific use of vermicompost for remediation of atrazine-contaminated soil.

## 2. Materials and Methods

### 2.1. Chemicals and Materials

The analytical standards for atrazine were obtained from Shanghai Yien Chemical Technology Co., Ltd. (Shanghai, China). The hydroxyatrazine (HYA) pure type, deethylatrazine (DEA), and deisopropylatrazine (DIA) analytical standards were of high-performance liquid chromatography (HPLC) grade and were obtained from Dr. Ehrenstorfer (Germany). The methanol and acetonitrile solvents were of HPLC grade and were obtained from Tianjin Kemiou Chemical Reagent Co., Ltd. (Tianjin, China). Other chemical reagents were analytical grade. In this experiment, the soil was collected from the 0–20 cm soil layer of the experimental base of Jilin Agricultural University in Changchun City, Jilin Province (43°26′ N, 125°05′ E), and atrazine was not detected in the soil. The collected soil was naturally air-dried and sieved through a 2 mm mesh. The vermicompost was prepared by mixing maize stover and cattle dung in equal proportions to prepare vermicompost, and the vermicompost was stored at −80 °C for 45, 60, and 75 days. Atrazine was not detected in the vermicompost. The physicochemical properties of the soil and vermicompost are shown in Table 1.

### 2.2. Atrazine Degradation Experiment

A total of eight treatments were designed for the biodegradation test of atrazine, as shown in Table 2. A portion of the original soil was sterilized at 121 °C for 3 h to prepare sterilized soil. The atrazine solution (100 mg/L in methanol) was added to 1500 g of soil, so that the initial content of atrazine was 10 mg/kg (dry weight), and evaporated at room temperature for 24 h to completely remove the methanol. In this study, the contamination level of atrazine was set to 10 mg/kg. Based on the concentration of atrazine detected in soil (3–6 mg/kg in most agricultural soils) and the concentration set by reference to other relevant studies (mostly 10 mg/kg) [17].

Reference to the amount of organic fertilizer applied to the farmland is 30–60 m^3^/ha^−1^ [31]. In this experiment, 50 mg of vermicompost was mixed per gram of soil. The reference for the application of organic fertilizer to agricultural land was 30–60 m^3^/ha [31], and, in this experiment, 50 mg of vermicompost was mixed per gram of soil. Sterile distilled water was added to the soil to maintain the maximum soil water-holding capacity at 60%. The biodegradation experiment was incubated at 25 °C for 40 days in the dark and destructively sampled at 0, 10, 20, 30, and 40 days. The collected samples were frozen at −20 °C and used for analyzing the physicochemical properties, atrazine, and atrazine metabolite content of the samples. In addition, the samples (about 20g) were taken on days 0, 20, 30, and 40, respectively, and stored at −80 °C for microbial community analysis. All treatments were replicated three times.

### 2.3. Chemical Analysis of Atrazine and Metabolites

To determine the concentrations of atrazine and three major metabolites (HYA, DEA, and DIA) in each sample, 5 g of lyophilized sample was taken from each treatment, and 5 g of anhydrous sodium sulfate was added to 20 mL of methanol extract, which was mixed thoroughly and then ultrasonicated for 15 min. The supernatant was collected after centrifugation at 8000 rpm for 10 min. A total of three extractions were carried out using fresh methanol and all the supernatants were combined and concentrated to 1 mL by rotary evaporator. The liquid was filtered through a 0.22 μm microporous membrane and then subjected to gas chromatography-mass spectrometry (GC-MS) and high-performance liquid chromatography (HPLC) analysis.

An ISQ-GC (Thermo Fisher, Waltham, MA, USA) equipped with an HP5-MS column (30 m × 0.25 mm × 0.25 µm) was used to detect the concentration of atrazine, DEA, and DIA. The operating conditions were as follows: temperature: 280 °C; injection volume: 1 µL; carrier gas: helium; flow rate: 1 mL/min; ion transfer line temperature: 280 °C; ion source: EI source at 280 °C; confirmed ions (*m*/*z*): atrazine: 173, 200, 215; DEA: 145, 172, 187; DIA: 145, 158, 173. Quantification ions (*m*/*z*): 200 (atrazine), 172 (DEA), 145 (DIA). The column temperature was maintained at 60 °C for 1 min, then increased to 260 °C at a rate of 15 °C/min and maintained at this temperature for 5 min. The recovery rate was 89.6–92.7% for atrazine, 93.7–95.4% for DEA, and 89.2–92.6% for DIA. High-performance liquid chromatography (HPLC, Waters Alliance E2695, Milford, MA, USA) equipped with CORTECS T3 Column (2.7 μm, 150 × 4.6 mm, Waters) was used to detect the concentration of HYA. The eluent A was acetonitrile (*v*/*v*) and eluent B was potassium dihydrogen phosphate solution (1 mmol/L). The gradient program was 30% A, 70% B for 15 min, 65% A, 35% B for 5 min, and 30% A, and 70% B for 10 min at a flow rate of 0.5 mL/min with a 10 μL injection volume. The column temperature was 25 ± 1 °C, and the detection wavelength was 220 nm. The recovery of HYA was 92.4–97.2%. The final concentrations of atrazine and its metabolites were corrected according to the recoveries.

### 2.4. Soil and Vermicompost Physicochemical Property Analysis

The samples were suspended in distilled water at a ratio of 1:2.5 (*m*/*m*), shaken at 120 rpm for 1 h, and filtered. The filtrate was then used to determine the pH value. Soil organic matter (SOM) was determined by the potassium dichromate oxidation method [32] (Nelson and Sommers, 1996). Soil total nitrogen (TN) was determined by the Kjeldahl method [33]. Humic acid (HA) and fulvic acid (FA) were extracted from soil and vermicompost using the previously reported methods [34,35].

### 2.5. DNA Extraction, Amplification, and Sequencing

DNA was extracted using the E.Z.N.A. Soil DNA Extraction Kit (OMEGA Biotek, Norcross, GA, USA) from 0.2 g of culture samples, with three replicates per treatment. The extracted DNA was detected by 1% agarose gel electrophoresis (*w*/*w*), and the concentration and purity of extracted DNA were determined by Nanodrop ultra-micro spectrophotometer (Thermo Fisher Scientific, Waltham, MA, USA). The bacterial V3–V4 region of 16S rRNA genes was amplified using primers V338F (5′-ACTCCTACGGGAGGCAGCAG-3′) and V806R (5′-GGACTACHVGGGTWTCTAAT-3′). All PCR reactions were performed according to the method of Luo et al. (2022) [17]. The purified PCR products were used for high-throughput sequencing. Sequencing was performed using the Illumina MiSeq PE250 platform by Shanghai Meiji Bio Biotech (Shanghai, China). The sequences were analyzed for microbial ecological quantification. All sequences were clustered into operational taxonomic units (OTUs) based on a 97% threshold identity by the SILVA database (https://www.arb-silva.de/) URL (accessed on 31 December 2022). A representative sequence from each OTU was selected for downstream analysis.

### 2.6. Statistical Analysis and Bioinformatics

Data analysis was performed using IBM SPSS Statistics for Windows (version 26.0) software. Differences in soil physicochemical properties, atrazine content, and its metabolites among treatments were analyzed at the *p* < 0.05 level of significance. Diversity and similarity analyses of soil bacterial community composition and LEfSe analysis were performed using R software (Version 2.15.3). Gephi software (v0.10.1) was used to construct microbial symbiotic network diagrams and microbial relationship diagrams with atrazine and metabolites based on Spearman correlation analysis. The correlation heat map of atrazine degradation rates and atrazine metabolites with soil physicochemical properties was constructed based on Pearson correlation analysis.

## 3. Results

### 3.1. Soil Physicochemical Properties

**Figure 1:**
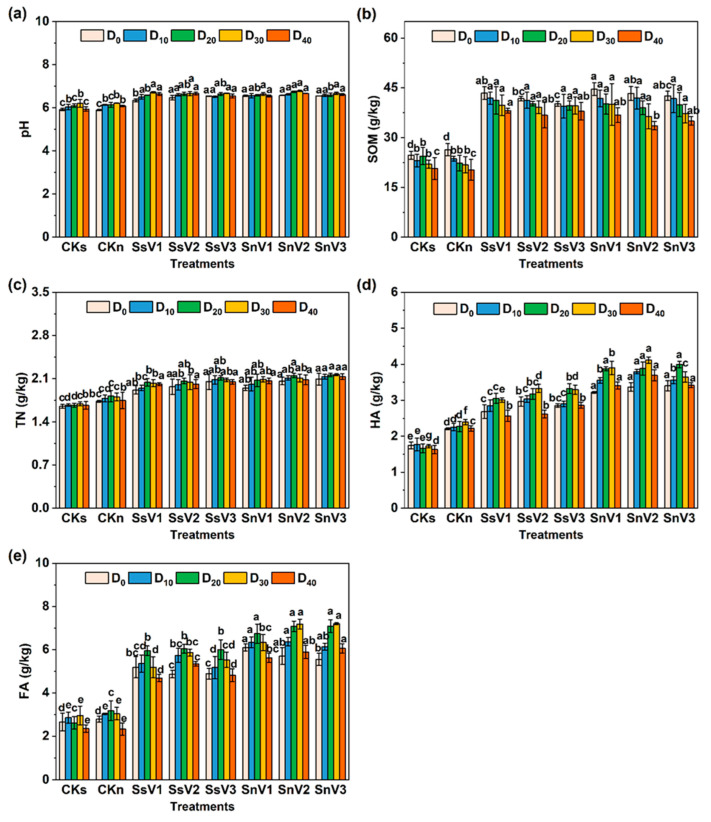
Soil physicochemical properties in different treatments. (**a**) pH; (**b**) SOM; (**c**)TN; (**d**) HA; (**e**) FA. CKs: sterilized soil. CKn: unsterilized soil. SsV1: sterilized soil amended with 45 days of vermicompost. SsV2: sterilized soil amended with 60 days of vermicompost. SsV3: sterilized soil amended with 75 days of vermicompost. SnV1: unsterilized soil amended with 45 days of vermicompost. SnV2: unsterilized soil amended with 60 days of vermicompost. SnV3: unsterilized soil amended with 75 days of vermicompost. D_0_: day 0 of atrazine degradation. D_10_: day 10 of atrazine degradation. D_20_: day 20 of atrazine degradation. D_30_: day 30 of atrazine degradation. D_40_: day 40 of atrazine degradation. SOM: soil organic matter. TN: total nitrogen. HA: humic acid. FA: fulvic acid. Data are means ± standard deviation from three replicates. Different letters indicate significant differences (Tukey’s test, *p* < 0.05) among treatments (*n* = 3).

The changes in pH, SOM, TN, HA, and FA in each treatment group during atrazine degradation are shown in Figure 1. The HA content of CKn was 2.21, 2.40, and 2.22 g/kg at days 0, 30, and 40, which were 26.29%, 38.73%, and 36.20% higher than that of CKs, respectively. This was because sterilized soil cannot decompose organic matter to produce humus through microbial activities, thus affecting the formation and accumulation of humic acid. Compared with CKs and CKn, the addition of vermicompost significantly (*p* < 0.05) increased the pH value, SOM, TN, HA, and FA content of the soil (Figure 1). In the sterilized soil amended with vermicompost, the HA and FA content of SsV2 reached a maximum of 3.33 and 6.04 g/kg at days 30 and 20, respectively, which were 92.49% and 130.53% higher than CKs. The TN content of SsV3 reached a maximum of 2.13 g/kg at day 20, which was 27.54% higher than CKs. In the unsterilized soil amended with vermicompost, the highest pH of 6.78 was recorded in SnV2. The addition of vermicompost increased soil SOM content by 62.84–80.75% at day 0 (Figure 1b), and the maximum SOM content of SnV1 was 44.62 g/kg. Meanwhile, the HA content of SnV2 reached a maximum of 4.11 g/kg for SnV2 at day 30, which was 71.97% higher than CKn. The TN and FA content of SnV3 reached a maximum at day 30 with 2.16 and 7.20 g/kg, respectively, which were 19.34% and 136.07% higher than CKn. Throughout the atrazine degradation process, the TN, HA, and FA content of each treatment group showed an increasing and then decreasing trend, except for CKs.

### 3.2. Effect of Vermicompost on the Degradation Performance and Metabolites of Atrazine

**Figure 2:**
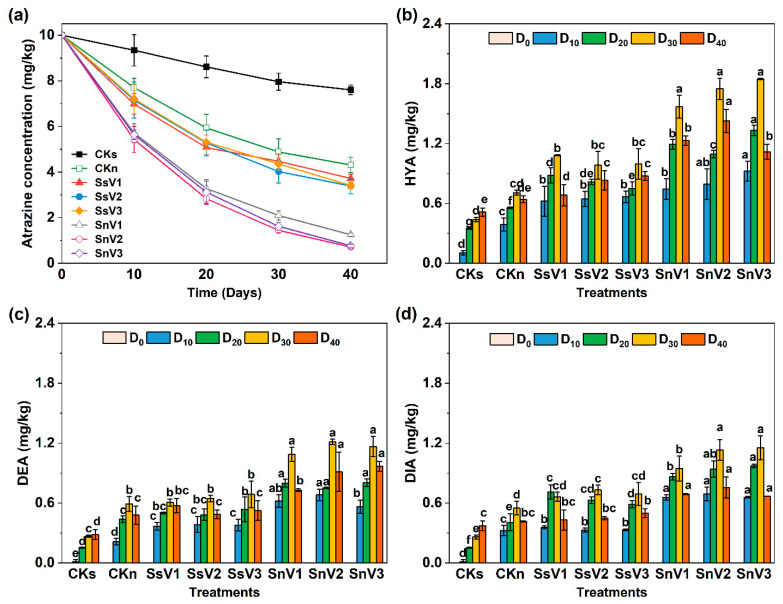
The contents of atrazine and three metabolites throughout the atrazine degradation process in different treatments. (**a**) Atrazine concentration; (**b**) HYA; (**c**) DEA; (**d**) DIA. CKs: sterilized soil. CKn: unsterilized soil. SsV1: sterilized soil amended with 45 days of vermicompost. SsV2: sterilized soil amended with 60 days of vermicompost. SsV3: sterilized soil amended with 75 days of vermicompost. SnV1: unsterilized soil amended with 45 days of vermicompost. SnV2: unsterilized soil amended with 60 days of vermicompost. SnV3: unsterilized soil amended with 75 days of vermicompost. D_0_: day 0 of atrazine degradation. D_10_: day 10 of atrazine degradation. D_20_: day 20 of atrazine degradation. D_30_: day 30 of atrazine degradation. D_40_: day 40 of atrazine degradation. HYA: hydroxyatrazine. DEA: deethylatrazine. DIA: deisopropylatrazine. Data are means ± standard deviation from three replicates. Different letters indicate significant differences (Tukey’s test, *p* < 0.05) among treatments (*n* = 3).

The concentration of atrazine decreased in all groups during soil incubation (Figure 2a). The degradation of atrazine in the different groups showed a rapid and then gradually slowed down degradation trend, and all of them followed the first-order kinetic equation (Table 3). After 40 days of biodegradation, the atrazine degradation rates were 24% and 56.9% for CKs and CKn, and the half-lives were 97.06 d and 29.85 d, respectively (Table 3). In CKs, soil humus and clay minerals catalyzed the hydrolysis of atrazine to form 2-hydroxyatrazine. In contrast, the degradation of atrazine in CKn was 32.90% higher than in CKs, which proved the presence of indigenous microorganisms and the active biodegradation of atrazine in the soil. In the sterilized soil amended with vermicompost, the atrazine degradation rates were 62.8%, 66.1%, and 65.9% in SsV1, SsV2, and SsV3, respectively, which were higher than CKs and CKn. The results showed that the microflora in vermicompost could enhance the biodegradation of atrazine, and the degradation ability was higher than that of soil. The degradation rate of atrazine was significantly higher in the unsterilized soil amended with vermicompost. The atrazine degradation rates of SnV1, SnV2, and SnV3 were 87.5%, 92.9%, and 92.3%, respectively. The half-lives of atrazine in each treatment group, in descending order, were CKs, CKn, SsV1, SsV3, SsV2, SnV1, SnV3, and SnV2 (Table 3), which demonstrated that vermicompost could enhance the degradation of atrazine, and the addition of vermicompost enhanced the microbial degradation of atrazine in soil.

Atrazine metabolite contents during soil incubation are shown in Figure 2b–d. The contents of the metabolites HYA, DIA, and DEA of CKs increased gradually and peaked at day 40, which were 0.61, 0.37, and 0.29 mg/kg, respectively. In CKn, the contents of HYA, DIA, and DEA all peaked at day 30, which were 0.85, 0.55, and 0.69 mg/kg, respectively, and were significantly higher (*p* < 0.05) than in CKs. The results showed that all three metabolic pathways of atrazine were present in both sterilized and unsterilized soils and that the degradation of atrazine by indigenous soil microorganisms was limited. The contents of HYA, DIA, and DEA were higher in the sterilized soil amended with vermicompost groups than in CKs and CKn from 0 to 40 days. At day 30, the HYA content of SsV1 reached a peak of 1.38 mg/kg. The highest DEA content of SsV3 was 0.79 mg/kg. The DIA content of SsV2 reached a peak of 0.83 mg/kg. This indicated that vermicompost could promote the decomposition of atrazine into HYA, DEA, and DIA. At day 40, HYA, DIA, and DEA content decreased in the sterilized soil amended with vermicompost groups. The contents of HYA, DIA, and DEA in the unsterilized soil amended with vermicompost groups peaked at day 30. Among them, SnV3 had the highest HYA and DIA contents at 1.85 and 1.16 mg/kg, respectively, which were 2.17 and 1.78 times higher than that of CKn, respectively. SnV2 had the highest DEA content at 1.21 mg/kg, which was 1.76 times higher than that of CKn. More atrazine metabolites were accumulated in the unsterilized soil amended with vermicompost groups than in the sterilized soil amended with vermicompost groups.

### 3.3. Soil Bacterial Community Diversity

**Figure 3:**
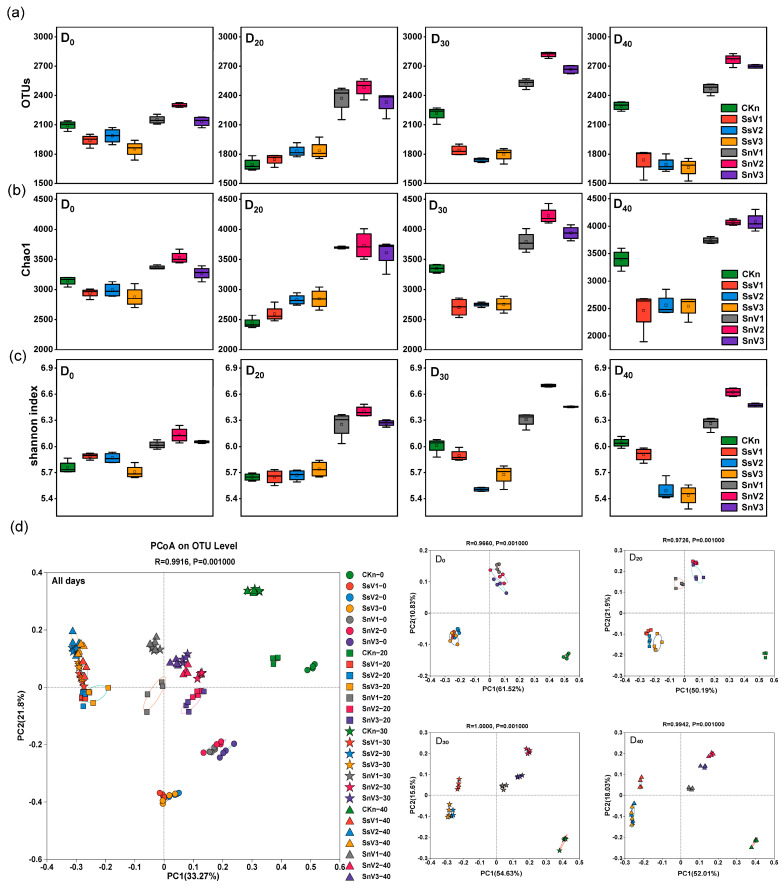
Dynamic changes in soil bacterial diversity in different treatments. (**a**) OTUs; (**b**) chao1; (**c**) Shannon’s index; (**d**) Principal coordinate analysis (PCoA) of bacterial community based on Bray–Curtis distance in different treatments, including total PCoA plots for all samples and PCoA plots for each time. CKs: sterilized soil. CKn: unsterilized soil. SsV1: sterilized soil amended with 45 days of vermicompost. SsV2: sterilized soil amended with 60 days of vermicompost. SsV3: sterilized soil amended with 75 days of vermicompost. SnV1: unsterilized soil amended with 45 days of vermicompost. SnV2: unsterilized soil amended with 60 days of vermicompost. SnV3: unsterilized soil amended with 75 days of vermicompost. D_0_: day 0 of atrazine degradation. D_10_: day 10 of atrazine degradation. D_20_: day 20 of atrazine degradation. D_30_: day 30 of atrazine degradation. D_40_: day 40 of atrazine degradation.

In this study, high-throughput sequencing was performed for all groups, and no bacterial community was detected in the CKs treatment. At the 97% sequence similarity level, the number of OTUs, Shannon’s index, and Chao1 from each group are shown in Figure 3a–c. At days 0, 30, and 40, the OTU numbers, Shannon’s index, and Chao1 of SsV1, SsV2, and SsV3 were lower than those of CKn, so the community richness and diversity of vermicompost were lower than CKn except on day 20. At day 0, the number of OTUs, Shannon’s index, and Chao1 of SnV1, SnV2, and SnV3 were higher than that of CKn. This indicated that vermicompost increased the richness and diversity of the soil community. At day 30, the OTU numbers, Shannon’s index, and Chao1 of SnV1, SnV2, and SnV3 continued to increase, and the OTU numbers, Shannon index, and Chao1 of SnV2 reached the highest. At day 40, the OTU numbers, Shannon index, and Chao1 continued to increase in CKn and SnV3, while the community richness and diversity decreased in SnV1 and SnV2, but both were greater than in CKn. SnV2 had the highest community richness and diversity throughout the degradation process. Principal Coordinate Analysis (PCoA) was used to compare the diversity of bacterial communities between groups, and the differences between communities were measured based on β-diversity (Figure 3d). Using Bray–Curtis differences, the first two principal components explained 55.07% of the community variance. Adonis analysis showed that there was a significant difference between the groups in the community (*p* = 0.001). From day 0, the bacterial community structure of CKn, the sterilized soil amended with vermicompost groups, and the unsterilized soil amended with vermicompost groups were separated, which indicated that the addition of vermicompost changed the bacterial community structure of the soil. At day 30, the bacterial community structure of SsV1 was separated from that of SsV2 and SsV3, and the bacterial community structure of SnV1 was separated from that of SnV2 and SnV3. At this time, the community difference between treatments was the largest. At day 40, the bacterial community structure of CKn, the sterilized soil amended with vermicompost groups, and the unsterilized soil amended with vermicompost groups were still significantly different. Compared to day 0, the bacterial community structure of the sterilized soil amended with vermicompost groups became more similar on days 20, 30, and 40. This indicated that there was little change in the community structure of the sterilized soil amended with vermicompost groups from day 20 to day 40. Meanwhile, the community structure of CKn and the unsterilized soil amended with vermicompost groups showed little change at days 30 and 40, which was different from the community structure at days 0 and 20. The bacterial community structure of SsV2 and SsV3 in the sterilized soil amended with vermicompost groups was closer (at days 30 and 40) than that of SsV1. The bacterial community structure of SnV2 and SnV3 in the unsterilized soil amended with vermicompost groups was closer (at days 20 and 40) than that of SnV1.

### 3.4. Soil Bacterial Community Composition

**Figure 4:**
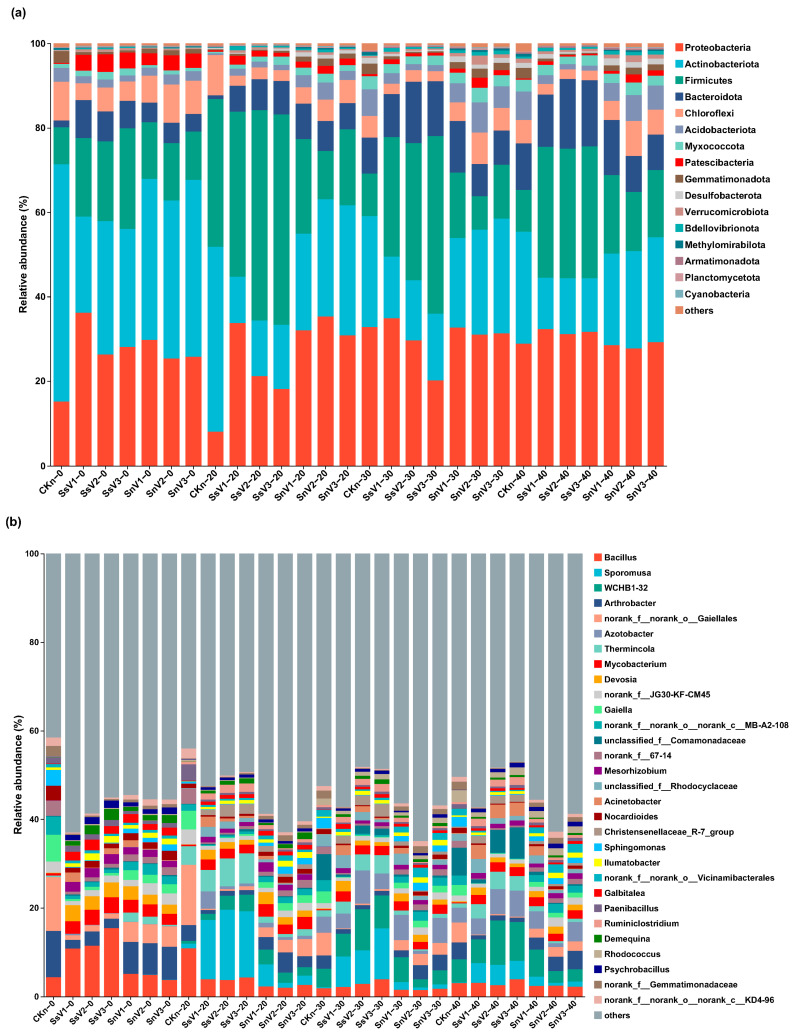

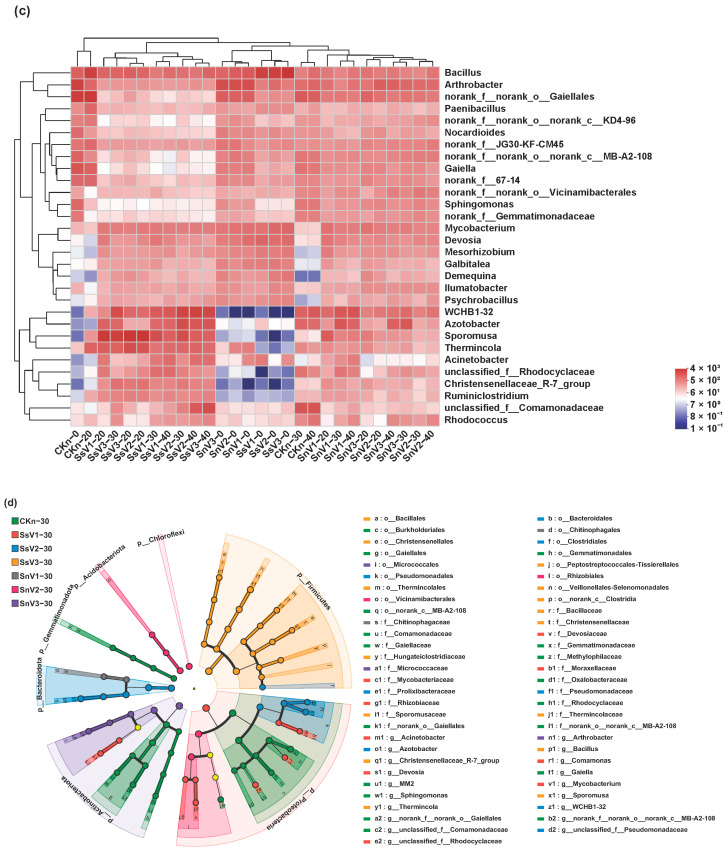
Changes in the relative abundance of bacterial community in different treatments. (**a**) Community structure of soil bacteria at phyla level in different treatments. (**b**) Community structure of soil bacteria at genera level in different treatments. (**c**) Community heatmap of soil bacteria at genera level in different treatments. (**d**) Results of LEfSe analysis among different treatments at bacterial taxonomic levels. CKs: sterilized soil. CKn: unsterilized soil. SsV1: sterilized soil amended with 45 days of vermicompost. SsV2: sterilized soil amended with 60 days of vermicompost. SsV3: sterilized soil amended with 75 days of vermicompost. SnV1: unsterilized soil amended with 45 days of vermicompost. SnV2: unsterilized soil amended with 60 days of vermicompost. SnV3: unsterilized soil amended with 75 days of vermicompost.

The relative abundance of bacteria in each treatment group is shown in Figure 4. At the phylum level (Figure 4a), the dominant phyla for each group were Proteobacteria, Actinobacteria, Firmicutes, Bacteroidota, and Chloroflexi, which accounted for 79–98% of the total number of bacteria. Proteobacteria was the most abundant phylum among all taxa, followed by Actinobacteria. At day 0, Actinobacteria was the most abundant phylum in CKn and the unsterilized soil amended with vermicompost groups. Proteobacteria was the most abundant phylum in the sterilized soil amended with vermicompost groups, followed by Actinobacteria. The addition of vermicompost increased the relative abundance of Proteobacteria, Firmicutes, Bacteroidota, and Patescibacteria in the soil. At day 20, the relative abundance of Proteobacteria decreased in the sterilized soil amended with vermicompost groups, and Firmicutes became the most abundant phylum, with 39.14%, 49.86%, and 49.82% for SsV1, SsV2, and SsV3, respectively. The relative abundance of Proteobacteria was the highest in the unsterilized soil amended with vermicompost groups, with 32.04%, 35.31%, and 30.85% for SnV1, SnV2, and SnV3, respectively. The relative abundance of Proteobacteria, Bacteroidota, and Patescibacteria in the unsterilized soil amended with vermicompost groups remained higher than in CKn. On day 30, Proteobacteria became the most abundant phylum in CKn, while the relative abundance of Bacteroidota and Acidobacteria increased in all groups compared to day 20. At day 40, Proteobacteria became the most abundant phylum again in the sterilized soil amended with vermicompost groups. The relative abundance of Firmicutes in the unsterilized soil amended with vermicompost groups was higher than that in CKn. The relative abundance of Proteobacteria, Firmicutes, and Bacteroidota in the sterilized soil amended with vermicompost groups was higher than that of CKn.

At the genus level (Figure 4b), at day 0, the dominant bacteria of CKn were norank_f_norank_o_*Gaiellales*, *Arthrobacter*, *Gaiella*, *Bacillus*, and norank_f_norank_o_norank_c_*MB-A2-108*, with relative abundances of 12.14%, 10.45%, 5.99%, and 4.38%, respectively. The dominant bacteria in the sterilized soil amended with vermicompost groups were *Bacillus*, *Devosia*, *Mycobacterium*, *Arthrobacter*, and *Mesorhizobium*. Among these, SsV3 had the highest relative abundance of *Bacillus*, *Devosia*, and *Mycobacterium*, which were 15.46%, 3.58%, and 3.31%, respectively. The dominant bacteria in the unsterilized soil amended with vermicompost groups were *Arthrobacter*, *Bacillus*, norank_f_norank_o_*Gaiellales*, *Mycobacterium*, and *Devosia*. The relative abundance of the above five dominant bacteria in SnV1 was the highest, which was 7.16%, 5.12%, 4.52%, 2.87%, and 3.07%, respectively. The dominant bacteria in CKn at day 20 changed from day 0 and included norank_f_norank_o_*Gaiellales*, *Bacillus*, *Thermincola*, *Gaiella*, and *Paenibacillus*. The community composition of the sterilized soil amended with vermicompost groups changed significantly. The dominant bacteria were *Sporomusa*, *Thermincola*, *Bacillus*, *WCHB1-32*, and *Mycobacterium*. The relative abundance of Sporomusa in SsV2 was the highest (15.88%), and the relative abundance of Thermincola in SsV3 was the highest (6.81%). The relative abundance of *Sporomusa* and *WCHB1-32* in the unsterilized soil amended with vermicompost groups was increased, and the dominant bacteria were *Arthrobacter*, norank_f_norank_o_*Gaiellales*, *Sporomusa*, *WCHB1-32*, and *Mycobacterium*. At day 30, the community composition of CKn changed significantly, and the dominant bacteria were unclassified_f_*Comamonadaceae*, norank_f_norank_o_*Gaiellales*, *WCHB1-32*, *Azotobacter*, and *Arthrobacter*. The relative abundance of *WCHB1-32* and *Azotobacter* increased in the sterilized soil amended with vermicompost groups, with the highest relative abundance of 0.93% and 0.75% in SsV2, respectively. *Azotobacter* became the most abundant genus in the unsterilized soil amended with vermicompost groups, and the relative abundance of *Azotobacter* in SnV1 was the highest (5.71%). The relative abundance of unclassified_f_*Comamonadaceae* and *WCHB1-32* in CKn increased at day 40 compared to day 30. The relative abundance of unclassified_f_*Comamonadaceae* in the sterilized soil amended with vermicompost groups increased, and the relative abundance of *Sporomusa* decreased compared with day 30. The relative abundance of *Arthrobacter* and *Bacillus* in the unsterilized soil amended with vermicompost groups increased compared with day 30.

Heat map analysis showed a significant separation of soil and vermicompost microbial communities, indicating that soil microbial community structure was significantly altered by vermicompost (Figure 4c). Heat map clustering results showed significant separation of microbial communities in the CKn, the sterilized soil amended with vermicompost groups, and the unsterilized soil amended with vermicompost groups. Consistent with the results obtained from PCoA, the bacterial communities of each group were more similar on days 30 and 40 compared to days 20 and 0. The bacterial communities of SsV2 and SsV3 were more similar to each other than to SsV1. Compared to SnV1, the bacterial communities of SnV2 and SnV3 were similar from days 20 to 40. Heat map analysis at the genus level for each group showed that from day 0 to 40, after the addition of vermicompost to unsterilized soil, the relative abundances of *Mycobacterium*, *Devosia*, *Mesorhizobium*, *Sporomusa*, *Ilumatobacter*, *Galbitalea*, *Demequina*, and *Psychrobacillus* increased from 0.51%, 0.12%, 0.05%, 1.03%, 0.56%, 0.1%, 0.03%, and 0.05% to 3.58%, 3.31%, 2.16%, 4.99%, 1.34%, 1.63%, 1.78%, and 1.50%, respectively. Notably, *Mesorhizobium*, *Demequina*, and *Psychrobacillus* were not found in CKn, which was introduced by vermicompost. The highest relative abundance of *Mycobacterium*, *Devosia*, *Mesorhizobium*, *Sporomusa*, and *Galbitalea* were found in SnV1. SnV3 had the highest relative abundance of *Ilumatobacter*, *Demequina*, and *Psychrobacillus*. Since metabolite content and bacterial community diversity peaked at day 30 in most groups, the samples of each group from day 30 were selected for LEfSe analysis to further investigate the differences in bacterial community composition among groups. As shown in Figure 4d, the results showed that all LDA values were greater than 4, indicating that there are significant differences at the taxonomic level. At the bacterial phylum level, all groups overlapped in Proteobacteria, Actinobacteria, Bacteroidota, and Firmicutes, indicating that the degradation of atrazine involves the participation of bacteria from different phyla. Gemmatimonadota was significantly enriched in CKn, while Firmicutes was significantly enriched in SsV3, and Acidobacteriota and Chloroflexi were enriched in SnV2. At the genus level, CKn-enriched genera were *Gaiella*, *MM2*, *Sphingomonas*, norank_f_norank_o_*Gaiellales*, norank_f_norank_o_norank_c_*MB-A2-108*, and unclassified_f_*Comamonadaceae*. The SsV1-enriched genera were *Acinetobacter*, *Comamonas*, *Devosia*, *Mycobacterium*, and g_unclassified_f_*Rhodocyclaceae*. *Azotobacter*, *WCHB1-32*, and unclassified_f_*Pseudomonadaceae* were enriched in SsV2. The genera enriched in SsV3 included *Bacillus*, *Christensenellaceae_R-7_group*, *Sporomusa*, and *Thermincola*, and *Arthrobacter* was significantly enriched in SnV3.

**Figure 5:**
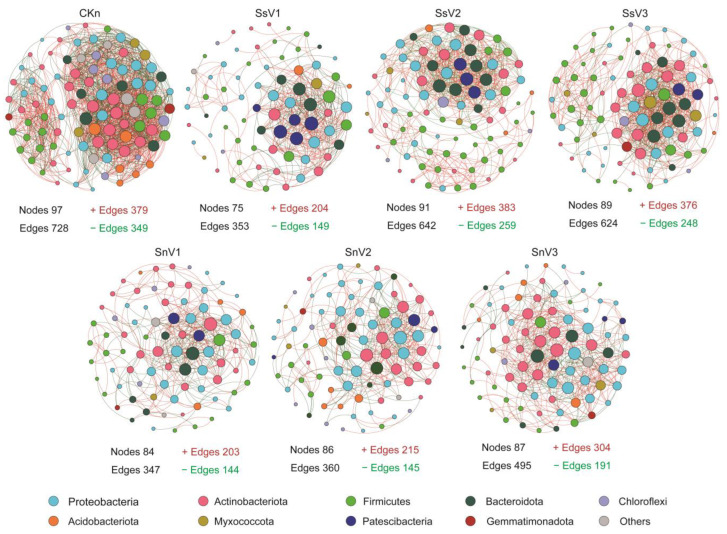
Network co-occurrence analysis of the bacterial community in different treatments. The connections stand for R_SPEARMAN_ < −0.8 (green edges) and >0.8 (red edges) and *p*-value < 0.01. Each node represents an affiliated phylum, and the size of each node is proportional to the number of connections (degree). CKs: sterilized soil. CKn: unsterilized soil. SsV1: sterilized soil amended with 45 days of vermicompost. SsV2: sterilized soil amended with 60 days of vermicompost. SsV3: sterilized soil amended with 75 days of vermicompost. SnV1: unsterilized soil amended with 45 days of vermicompost. SnV2: unsterilized soil amended with 60 days of vermicompost. SnV3: unsterilized soil amended with 75 days of vermicompost.

The complexity of soil microbiota interaction in each group was explored through co-occurrence network analysis. Based on the 16S rRNA data from all samples, seven co-occurrence network diagrams (|r| > 0.8, *p* < 0.01) were constructed for the top 100 genera of microbial groups in different groups (Figure 5). The topological properties of the resulting networks were calculated to identify differences between the samples. As shown in Figure 5 and Table S1, compared with CKn, the network edges, average clustering coefficients, and average degrees in each group decreased. It indicated that vermicompost reduced the complexity of the network. However, compared with CKn (0.392), the modularity of SnV1 and SnV2 increased to 0.449. Additionally, soil microorganisms may work synergistically in atrazine degradation, and the topological roles of each genus in microbial networks were determined by RMT-based network analysis.

In CKn, three of the five key genera belonged to Actinobacteria (norank_f_*67-14*, norank_f_norank_o_*Gaiellales*, and unclassified_f_*Micromonosporaceae*), while the others belonged to Bacteroidota (*Puia*) and Nitrospirota (*Nitrospira*). In SsV1, three of the five key genera belonged to Patescibacteria (unclassified_o_*Saccharimonadales*, *TM7a*, and norank_f_*LWQ8*), while two belonged to Bacteroidota (*Flavobacterium* and norank_f_*Paludibacteraceae*). Three of the five key genera in SsV2 belonged to Patescibacteria (norank_f_*LWQ8*, unclassified_o_*Saccharimonadales*, and *TM7a*), while two belonged to Bacteroidota (*WCHB1-32* and norank_f_Bacteroidetes_*vadinHA17*). Three of the five key genera in SsV3 belonged to Bacteroidota (norank_f_Bacteroidetes_*vadinHA17*, *WCHB1-32*, and norank_f_*Saprospiraceae*), while the others belonged to Actinobacteria (*Nocardioides*) and Proteobacteria (*Azotobacter*). Two of the five key genera in SnV1 belonged to Bacteroidota (norank_f_Bacteroidetes_*vadinHA17* and *WCHB1*-*32*), while the others belonged to Actinobacteria (norank_f_norank_o_*OPB41*), Firmicutes (*Hydrogenispora*), and Patescibacteria (unclassified_o_*Saccharimonadales*). Two of the five key genera in SnV2 belonged to Actinobacteria (*Mycobacterium* and *Microbacterium*), while the others belonged to Proteobacteria (norank_f_norank_o_*R7C24*), Bacteroidota (*Flavobacterium*), and Patescibacteria (unclassified_o_*Saccharimonadales*). In SnV3, two of the five key genera belonged to Bacteroidota (norank_f_Bacteroidetes_*vadinHA17* and *Flavobacterium*), two belonged to Actinobacteria (*Microbacterium* and *Rubrobacter*), and the other belonged to Proteobacteria (*Paracoccus*). Actinobacteria had the largest percentage (28.28%) in CKn network, while Firmicutes had the largest percentage in SsV1, SsV2 and SsV3, which were 28%, 31.87 and 31.46%, respectively. Proteobacteria had the largest percentage in SnV1, SnV2, and SnV3, with 29.76%, 34.88%, and 31.03%, respectively. In addition, Patescibacteria were not found in the CKn network and only existed in the vermicompost groups.

### 3.5. Effect of Vermicompost on Soil Microbial Function

**Figure 6:**
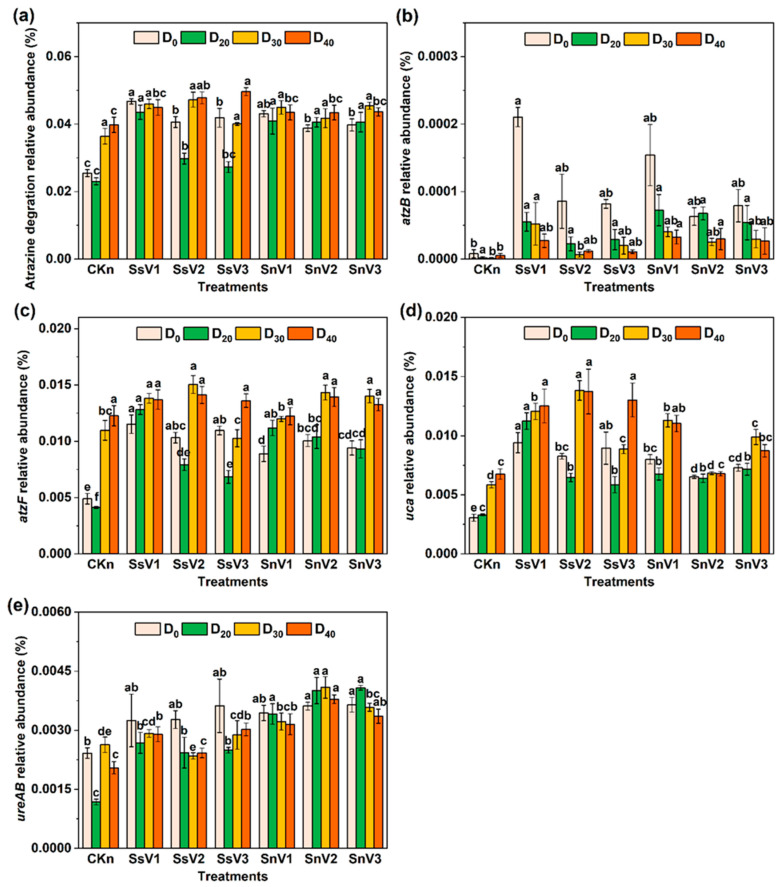
The relative abundance of atrazine metabolic pathway and functional genes predicted by PICRUSt2. (**a**) Atrazine degration; (**b**) *atzB*; (**c**) *atzF*; (**d**) *uca*; (**e**) *ureAB*. CKs: sterilized soil. CKn: unsterilized soil. SsV1: sterilized soil amended with 45 days of vermicompost. SsV2: sterilized soil amended with 60 days of vermicompost. SsV3: sterilized soil amended with 75 days of vermicompost. SnV1: unsterilized soil amended with 45 days of vermicompost. SnV2: unsterilized soil amended with 60 days of vermicompost. SnV3: unsterilized soil amended with 75 days of vermicompost. D_0_: day 0 of atrazine degradation. D_10_: day 10 of atrazine degradation. D_20_: day 20 of atrazine degradation. D_30_: day 30 of atrazine degradation. D_40_: day 40 of atrazine degradation. *atzB*: N-ethylaminohydrolase; *atzF*: allophanate hydrolase; *ura*: urea carboxylase; *ureAB*: urease subunit gamma/beta; Different letters indicate significant difference (*p* < 0.05).

The relative abundance of the atrazine degradation pathway (00791, KEGG pathway database number) [36] was selected from PICRUSt2 to analyze the effect of atrazine degradation on microbial function (Figure 6). The relative abundance of atrazine degradation was higher in the vermicompost groups than in CKn, and it decreased at day 20 compared to day 0 in all groups except SnV2 and SnV3. In the unsterilized soil amended with vermicompost groups, SnV3 had the highest relative abundance of atrazine degradation at day 30. In the sterilized soil amended with vermicompost, the relative abundance of atrazine degradation in SsV3 reached the highest of 0.050% at day 40. In the atrazine degradation pathway map (map 00791, Figure S1), the relative abundance of *atzB* (K03382, hydroxydechloroatrazine ethylaminohydrolase gene), *atzF* (K01457, allophanate hydrolase gene), *uca* (K01941, urea carboxylase gene), and *ureAB* (K14048, urease subunit gamma/beta gene) were significantly different in each group (Figure 6). At days 0–20, the relative abundance of *atzB*, *atzF*, *uca*, and *ureAB* in the vermicompost-amended groups was significantly higher than that of CKn. Except for CKn, SsV2, and SnV2, the relative abundance of *atzB* showed a decreasing trend from day 0 to day 40, and the relative abundance of *atzB* in SsV1 was the highest on day 0. In the sterilized soil amended with vermicompost groups, the relative abundance of *atzF* and *uca* was highest at day 30 in SsV2, and *ureAB* was highest at day 0 in SsV3. In the unsterilized soil amended with vermicompost groups, the relative abundance of *atzF* and *ureAB* was highest at day 30 in SnV2, while *uca* was highest at day 30 in SnV1.

### 3.6. Respective Correlations Between Degradation Rate and Metabolites of Atrazine and Soil Bacteria, and Soil Physicochemical Properties 

**Figure 7:**
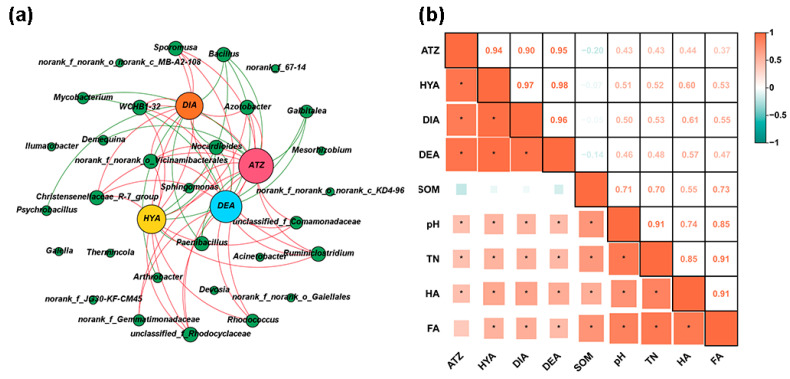
Co-occurrence network (**a**) of soil microbes, atrazine degradation rates, and atrazine metabolites are based on Spearman’s correlation (*p* < 0.01). Correlation heatmap (**b**) of atrazine degradation rates, atrazine metabolites, and soil physicochemical properties are based on Pearson’s correlation (*p* < 0.05). Significance levels are denoted with * *p* < 0.05. ATZ: atrazine degradation rate. HYA: hydroxyatrazine. DEA: deethylatrazine. DIA: deisopropylatrazine. SOM: soil organic matter. TN: total nitrogen. HA: humic acid. FA: fulvic acid.

Co-occurrence patterns between the soil bacteria and the atrazine degradation rates (ATZ) and its metabolites (HYA, DIA, and DEA) were visualized as a correlation network (Figure 7a), and co-occurrence network was based on Spearman correlation (*p* < 0.01). The network consisted of 34 nodes (30 genera, the atrazine degradation rate, and 3 metabolites) and 57 edges. There were more positive correlations (11) between soil bacteria and ATZ than negative correlations (6), and there were more positive correlations (9 with HYA, 7 with DIA, 10 with DEA) between soil bacteria and atrazine metabolites than negative correlations (4 with HYA, 5 with DIA, 5 with DEA). Bacteria significantly positively correlated (r > 0.5, *p* < 0.01) with ATZ included *WCHB1-32* and *Azotobacter*. Bacteria significantly positively correlated (r > 0.5, *p* < 0.01) with HYA included *Azotobacter* and *Christensenellaceae_R-7_group*. Bacteria significantly positively correlated (r > 0.5, *p* < 0.01) with DIA included *Sporomusa*, *Azotobacter*, and *Christensenellaceae_R-7_group*. Bacteria significantly positively correlated (r > 0.5, *p* < 0.01) with DEA included *Azotobacter*. Among them, *Azotobacter* showed a significant positive correlation (r > 0.5, *p* < 0.01) with ATZ and all three metabolites. *Azotobacter* showed the strongest correlation with ATZ (r = 0.65), followed by HYA (r = 0.61). *Bacillus* and *Paenibacillus* were significantly negatively correlated with ATZ and the three metabolites (r < −0.5, *p* < 0.01). A heat map of the correlation of ATZ, atrazine metabolites, and soil physicochemical properties based on Pearson’s correlation is shown in Figure 7b, ATZ, HYA, DIA, and DEA showed a significant positive correlation (*p* < 0.05) with pH, TN, and HA, respectively. Among them, ATZ, HYA, DIA, and DEA showed the highest correlation with HA. Additionally, HYA and DIA had stronger connections with HA (r = 0.60, 0.61) and FA (r = 0.53, 0.55) than TN (r = 0.52, 0.53) and pH (r = 0.51, 0.50), whereas DEA was more correlated with HA (r = 0.57) and TN (r = 0.48) than FA (r = 0.47) and pH (r = 0.46).

## 4. Discussion

This study demonstrated that indigenous soil microorganisms in the soil played an essential role in the degradation of atrazine. The atrazine degradation rate of CKn (56.9%) was higher than that of CKs (24%). The half-life of atrazine in CKs was 3.25 times longer than that of CKn, indicating that the atrazine degradation in soil depended on microbial catabolism, which was similar to the results of previous studies. Liu et al. (2023) [37] reported that the degradation rate of atrazine in unsterilized soil was 98.41–99.43%, while in sterilized soil, it was only 22.53–66.50%, and the half-life of atrazine in sterilized soil was 3.6–71.4 times longer than that in unsterilized soil. Yale et al. (2017) [38] also reported that the half-life of atrazine in sterilized soil was 107.7 days, while, in unsterilized soil, it was only 20.4 days. Several soil microorganisms have been reported to be involved in atrazine degradation, including *Streptomyces*, *Massilia*, *Sphingomonas*, and *Nocardioides* [39]. Although soil microorganisms can degrade atrazine, the degradation effect is unsatisfactory, and only about 35.2–35.7% of atrazine in the soil can be degraded [17,40]. Vermicompost, as an efficient organic fertilizer, can provide sufficient nutrients while introducing new microorganisms to diversify the soil microbial community and accelerate the degradation of pollutants [17]. In this study, after 40 days of degradation, the degradation rate of atrazine in the sterilized soil amended with vermicompost group was 62.8–66.1%. Therefore, vermicompost had a degradation effect on atrazine and was stronger than soil. The degradation rate of atrazine in unsterilized soil amended with vermicompost groups was significantly increased. The degradation rate of atrazine was 87.5–92.9% at day 40. This result was consistent with previous research results that the addition of organic matter or waste can enhance the degradation of atrazine in soil [17].

In this study, the addition of vermicompost significantly increased soil pH, as well as the contents of SOM, TN, HA, and FA (Figure 1). It has been shown that the half-life of atrazine in acidic soils is significantly lower than that in alkaline soils, probably due to the easy hydrolysis of atrazine in acidic soils [37]. This finding contrasted with the results of this experiment. After adding vermicompost to the soil, the pH value increased to 6.34–6.78, which was close to the neutral pH, and the degradation rate of atrazine was increased. This may be because neutral pH can support higher microbial biomass and enzyme activity, which is beneficial to the biodegradation of atrazine [41]. In addition, studies have also shown that the degradation rate of atrazine was slower at pH < 5.5, and the degradation rate was significantly accelerated at higher pH [42]. SOM, HA, and FA are also the key factors influencing the retention, transport, and degradation of herbicides. SOM has been reported to play a dominant role in the adsorption and transport of organic pollutants in soil [43]. It is possible to increase herbicide adsorption by providing more adsorption sites for hydrogen bonding, carboxyl and phenolic amide groups, and hydrophobic bonding [44]. However, at the same time, SOM can adsorb microbial enzymes to form humic enzyme complexes, which are more stable than free enzymes to hydrolyze atrazine into HYA [45]. In addition, SOM can be used as a carbon and energy source to increase the number and activity of soil microorganisms, thus facilitating the biodegradation of herbicides [44]. Soil humus can also promote the removal of atrazine [17]. HA and FA contain acidic functional groups, which can accelerate atrazine degradation by catalyzing the hydroxylation of chlorine-1,3,5-triazine [46] and chlorine-s-triazine [47].

Soil microorganisms are another crucial factor in the remediation of organic pollutants. Vermicompost increased the diversity and richness of soil microbial communities. During the degradation process, the community richness of SnV2 was the highest. The dominant phyla in the soil were Actinobacteria and Proteobacteria, and the addition of vermicompost increased the relative abundance of Proteobacteria, Firmicutes, Bacteroidota, and Patescibacteria. Meanwhile, LEfSe analysis showed that Gemmatimonadota was significantly enriched in CKn, Firmicutes was significantly enriched in SsV3, and Acidobacteriota and Chloroflexi were enriched in SnV2. Studies have shown that Proteobacteria and Actinobacteria are widely distributed in the environment and are highly active in promoting plant growth, nutrient cycling, biocontrol, and pollutant degradation [48]. Firmicutes, Bacteroidota, Patescibacteria, Acidobacteriota, and Chloroflexi, have also been reported as dominant bacteria for the potential degradation of atrazine, which plays an important role in atrazine degradation. [40,49,50]. Some of the dominant genera in soil, such as *Arthrobacter*, *Gaiella*, *Bacillus*, and *Paenibacillus*, have been identified in previous studies as degraders of pesticides [51]. For example, *Arthrobacter* can grow with atrazine as the sole nitrogen source and convert atrazine to cyanuric acid with the help of hydrolytic enzymes [19]. It also accelerated the removal of atrazine from soil and plants, increased plant chlorophyll content, and regulated the transcriptome to reduce atrazine-induced oxidative stress in plants [16]. *Bacillus* promoted a high level of expression of atrazine-related degradation genes by using atrazine as the sole carbon source [18]. It was also able to degrade pesticides such as simazine, cycloheximide, and atrazine [52]. *Gaiella* has been considered a potential sulfadiazine-degrading bacterium and plays a dominant role in sulfadiazine degradation [53]. *Paenibacillus* could promote soil nutrient enhancement and the degradation of organic pollutants [54]. Therefore, the presence of these pesticides and pollutant-degrading bacteria is consistent with the higher atrazine degradation rate in CKn than that in CKs (Figure 2). The relative abundance of *Mycobacterium*, *Devosia*, *Mesorhizobium*, *Sporomusa*, *Ilumatobacter*, *Galbitalea*, *Demequina*, and *Psychrobacillus* increased after adding vermicompost to unsterilized soil, and the degradation rate of atrazine was 30.4%-36.0% higher than in CKn. Among them, *Sporomusa*, *Mycobacterium*, *Devosia*, and *Mesorhizobium* were also the dominant bacteria in the sterilized soil amended with vermicompost groups. Additionally, LEfSe analysis showed that *Devosia* and *Mycobacterium* were significantly enriched in the SsV1, *Azotobacter*, and *WCHB1-32* in SsV2, *Bacillus*, *Sporomusa*, and *Thermincola* in SsV3, and *Arthrobacter* in SnV3. Most of these bacteria are involved in organic pollutant degradation. For example, *Mycobacterium* was found to carry *atzB*, *atzC*, and *atzD* functional genes, which are involved in the dealkylation and dechlorination of atrazine. It played an important role in atrazine metabolism and degraded other triazine herbicides [15,55]. *Devosia* was identified as the core microorganism for the synergistic degradation of total petroleum hydrocarbons [56]. *Microbacterium* was able to degrade the organophosphorus pesticide chlorpyrifos by regulating the activities of glutathione s-transferase, catalase, and superoxide dismutase [57]. The abundance of *Sporomusa* in anaerobic sludge was positively correlated with the degradation capacity of acetochlor and played an important role in the degradation of acetochlor [58]. It has been reported that the removal of decabromodiphenyl ether in soil may be related to the enrichment of *Ilumatobacter* [59]. *Demequina* was a potentially beneficial bacterium in the intestine of crayfish under lead contamination [60]. *Psychrobacillus* could use petroleum as a source of energy and carbon; therefore, it has a potential role in removing petroleum contaminants [61]. In summary, vermicompost could enrich a variety of potential atrazine-degrading bacteria (*Mycobacterium*, *Devosia*, *Sporomusa*, *Ilumatobacter*, and *Galbitalea*) in the soil and introduce new microorganisms (*Mesorhizobium*, *Demequina*, and *Psychrobacillus*) into the soil to accelerate the biodegradation of atrazine.

Vermicompost reduced the complexity of the soil microbial co-occurrence network (Figure 5), with the number of positive correlations larger than that of negative correlations, and the modularity index increased. The addition of vermicompost makes the synergistic effect of microorganisms on atrazine simpler and more stable. Most of the key bacteria in CKn were Actinobacteria, whereas most of the key genera in the sterilized soil amended with vermicompost groups were Bacteroidota and Patescibacteria, and most of the key genera in the unsterilized soil amended with vermicompost groups were Actinobacteria and Bacteroidetes. Thus, vermicompost changed the key microorganisms in the soil. Vermicompost also changed the composition and abundance of soil microorganisms. Actinobacteria was the most abundant in the CKn co-occurrence network, Firmicutes was the most abundant in the sterilized soil amended with vermicompost groups, and Proteobacteria was the most abundant in the unsterilized soil amended with vermicompost groups. This may be due to the production of heat-resistant spores by genera such as *Bacillus*. Patescibacteria was present only in the vermicompost groups and not in CKn. Studies have shown that Patescibacteria plays an important role in the remediation of pollution caused by heavy metals, PAHs, and antibiotic-resistant bacteria [62,63,64]. Unclassified_o_*Saccharimonadales* in Patescibacteria was the key bacterium in SsV1, SsV2, SnV1, and SnV2, while norank_f_Bacteroidetes_*vadinHA17* and *WCHB1-32* in Bacteroidota were the key bacterium in SsV1, SsV2, and SnV1. This indicated that unclassified_o_*Saccharimonadales*, norank_f_Bacteroidetes_*vadinHA17*, and *WCHB1-32* have important synergistic effects in the co-occurrence network. The PICRUSt2 results further showed that vermicompost enhanced the metabolic function of atrazine and increased the relative abundance of the *atzB*, *atzF*, *uca*, and *ureAB* (Figure 6). Studies have reported that *atzB* carried by *Arthrobacter* and *Bacillus* was able to participate in the step of hydroxy atrazine decomposition to N-Isopropylammelide. It was an important step in the synthesis of cyanuric acid, an intermediate product of atrazine [19,20]. The *atzF* was the final step in the pathway of atrazine degradation, which completely mineralized atrazine into carbon dioxide and ammonia.

The biodegradation pathway of atrazine primarily includes hydrolysis and dealkylation. The hydrolysis process can also be referred to as the dechlorination process. During the hydrolysis reaction, the chlorine atom on atrazine is replaced by a hydroxyl group, converting it into hydroxyatrazine (HYA) [2]. DEA and DIA are produced through N-dealkylation of the side chains of atrazine [2]. In the unsterilized soil amended with vermicompost groups, the accumulation of HYA, DIA, and DEA was significantly higher than that of CKn, and HYA was significantly higher than DIA and DEA (Figure 2). This indicated that the addition of vermicompost promoted atrazine degradation through all three metabolic pathways, with dechlorination hydrolysis being the main metabolic pathway in soil. Organic fertilizers have been reported to enhance the degradation pathways of pesticides in soil, as evidenced by the increased accumulation of HYA, DIA, and DEA in atrazine-contaminated soil amended with sheep manure [65]. In this study, *Azotobacter* was significantly and positively correlated (r > 0.5, *p* < 0.01) with HYA, DIA, and DEA, indicating that *Azotobacter* was a key participant in the atrazine degradation process. It has been reported that the *Azotobacter chroococcum* strain can grow on atrazine as a carbon source, and the degradation rate of atrazine is above 77% [66]. In addition, HYA, DIA, and DEA were positively correlated with pH, HA, TKN, and FA, as shown by heat map analysis (Figure 7b). It has been reported that atrazine was degraded mainly by chemical transformation or simultaneous chemical and microbial transformation in acidic soils. Microbial degradation of atrazine produces DEA and DIA as the main metabolites, while HYA is produced by the chemical degradation of atrazine [67]. However, it has also been reported that the hydrolysis and dechlorination of atrazine by the microbial community are mediated by bacterial enzymes rather than by chemical hydrolysis [68]. Therefore, in weakly acidic soils, both pathways can lead to the degradation of atrazine to hydroxyatrazine. Additionally, some studies have shown that maize stover compost can increase the accumulation of hydroxyatrazine in soil, probably due to the high enzyme activity of its active microorganisms that degrade atrazine to hydroxyatrazine [69]. These studies were similar to these experimental conditions, thus explaining the higher accumulation of HYA than DIA and DEA in this study. Therefore, the addition of vermicompost can significantly promote the HYA, DIA, and DEA pathways by increasing soil pH, TN, HA, and FA, resulting in the production of more HYA, DIA, and DEA. In this study, the addition of vermicompost improved the physicochemical properties of soil, increased the abundance of native soil microorganisms, and introduced additional atrazine-degrading bacteria to enhance the relative abundance of atrazine degradation genes, thereby promoting three metabolic pathways and accelerating the atrazine degradation process.

## 5. Conclusions

In this study, sterilized soil, unsterilized soil, and the addition of vermicompost with different maturities were used to remediate atrazine contamination. Vermicompost was able to increase the degradation of atrazine in soil. SnV2 had the highest atrazine degradation rate of 92.9%. The degradation rate of atrazine in CKn was 56.9%. It was 32.9% higher than that of CKs. This shows that there are indigenous microorganisms that can degrade atrazine in the soil. The degradation rates of SsV1, SsV2, and SsV3 were higher than that of CKn; therefore, vermicomposting was able to introduce microorganisms capable of degrading atrazine. The pH, SOM, TN, HA, and FA contents were significantly increased after the vermicompost was amended to the soil. Compared to DEA and DIA, vermicompost promoted atrazine metabolism to produce more HYA. Additionally, vermicompost accelerated atrazine mineralization by enhancing the synergy among indigenous atrazine-degrading bacteria (e.g., *Mycobacterium*, *Devosia*, *Sporomusa*) and introducing new degraders (e.g., *Mesorhizobium*, *Demequina*). This study showed that all three maturities of vermicompost increased the degradation rate of atrazine, and 60 days of vermicompost had the greatest degradation effect on atrazine.

## Figures and Tables

**Table 1:** Physical and chemical properties of the experimental material.

Material	pH	TOC (g/kg)	TN (g/kg)	HA (g/kg)	FA (g/kg)
Soil	5.92 ± 0.11	14.87 ± 1.86	1.73 ± 0.08	1.81 ± 0.09	2.79 ± 0.15
45 days of vermicompost	7.61 ± 0.27	396.57 ± 14.84	15.85 ± 0.58	35.94 ± 1.04	33.24 ± 1.02
60 days of vermicompost	7.78 ± 0.32	381.62 ± 12.73	18.23 ± 0.75	39.75 ± 1.13	31.55 ± 1.23
75 days of vermicompost	7.56 ± 0.21	372.23 ± 10.14	19.24 ± 0.92	42.12 ± 0.97	29.86 ± 1.14

TOC: total organic carbon; TN: total nitrogen; HA: humic acid; FA: fulvic acid.

**Table 2:** Experimental treatments.

Treatment	Design	Soil (kg)	Atrazine Mass Fraction (mg/kg)
CKs	Sterilized soil	1.5	10
CKn	Unsterilized soil	1.5	10
SsV1	Sterilized soil + 45 days of vermicompost	1.5	10
SsV2	Sterilized soil + 60 days of vermicompost	1.5	10
SsV3	Sterilized soil + 75 days of vermicompost	1.5	10
SnV1	Unsterilized soil + 45 days of vermicompost	1.5	10
SnV2	Unsterilized soil + 60 days of vermicompost	1.5	10
SnV3	Unsterilized soil + 75 days of vermicompost	1.5	10

**Table 3:** Degradation dynamics of atrazine in different treatments.

Treatments	First-Order Kinetic Model	Half-Value Period	R^2^
CKs	$Ct=9.99e^{\left(-0.0071x\right)}$	97.06 ± 3.35 a	0.992
CKn	$Ct=9.83e^{\left(-0.0228x\right)}$	29.85 ± 4.67 b	0.983
SsV1	$Ct=9.66e^{\left(-0.0272x\right)}$	24.24 ± 2.42 c	0.958
SsV2	$Ct=9.84e^{\left(-0.0293x\right)}$	23.22 ± 3.75 c	0.989
SsV3	$Ct=9.82e^{\left(-0.0282x\right)}$	24.00 ± 2.39 c	0.989
SnV1	$Ct=9.95e^{\left(-0.0534x\right)}$	12.79 ± 1.08 d	0.998
SnV2	$Ct=10.04e^{\left(-0.0633x\right)}$	11.00 ± 0.97 d	0.999
SnV3	$Ct=10.05e^{\left(-0.0595x\right)}$	11.73 ± 0.38 d	0.998

CKs: sterilized soil. CKn: unsterilized soil. SsV1: sterilized soil amended with vermicompost were prepared for 45 days. SsV2: sterilized soil amended with vermicompost were prepared for 60 days. SsV3: sterilized soil amended with vermicompost were prepared for 75 days. SnV1: unsterilized soil amended with vermicompost were prepared for 45 days. SnV2: unsterilized soil amended with vermicompost were prepared for 60 days. SnV3: unsterilized soil amended with vermicompost were prepared for 75 days. Different letters indicate significant differences (Tukey’s test, p < 0.05) among treatments (*n* = 3).

## Data Availability

The datasets used or analyzed during the current study are available from the corresponding author upon reasonable request.

## Supplementary Materials

The following supporting information can be downloaded at https://www.mdpi.com/article/10.3390/toxics13010030/s1, Figure S1: The atrazine degradation pathway map in KEGG pathway (map 00791); Table S1: Topological characteristics of microbial network in different treatments.
