# Supplementary material for: Influence Mechanism of Vermicompost with Different Maturity on Atrazine Catabolism and Bacterial Community
Source: Toxics. 2025 Jan 1;13(1):30. doi: 10.3390/toxics13010030 (PMC11769362; doi:10.3390/toxics13010030)
Supplement: Supplementary file 1 [file toxics-13-00030-s001.zip › toxics-3377938-supplementary.pdf]

Supplementary material

Influence mechanism of vermicompost with different maturity on atrazine catabolism and bacterial community

Luwen Zhang[1], Lixin Xu[2], Zunhao Zhang[3], Jiaolin Li[1], Limeng Ren[1], Zhichen Liu[1], Yan Zhang[3], Yuxiang Chen[1,*]

[1]College of Biological and Agricultural Engineering, Jilin University, Changchun 130022, China;

[2]College of Life Sciences, Jilin University, Changchun 130012, China;

[3] The Electron Microscopy Center, Jilin University, Changchun 130000, China

[4]Costal Research and Extension Center, Mississippi State University, Pascagoula, MS 39567, United States

*Correspondence to: chen@jlu.edu.cn

Table S1 Topological characteristics of microbial network in different treatments.

Treatments	CKn	SsV1	SsV2	SsV3	SnV1	SnV2	SnV3
Total nodes	97	75	91	89	84	86	87
Total edges	728	353	642	624	347	360	495
Modularity	0.392	0.315	0.387	0.319	0.449	0.449	0.379
Average path distance	2.231	3.423	3.102	3.596	3.99	3.554	2.428
Average clustering coefficient	0.722	0.571	0.634	0.638	0.565	0.592	0.606
Average degree	29.30	9.41	14.11	14.02	8.26	8.37	11.38

CKs: sterilized soil. CKn: unsterilized soil. SsV1: sterilized soil amended with 45 days of vermicompost. SsV2: sterilized soil amended with 60 days of vermicompost. SsV3: sterilized soil amended with 75 days of vermicompost. SnV1: unsterilized soil amended with 45 days of vermicompost. SnV2: unsterilized soil amended with 60 days of vermicompost. SnV3: unsterilized soil amended with 75 days of vermicompost.

Figure S1

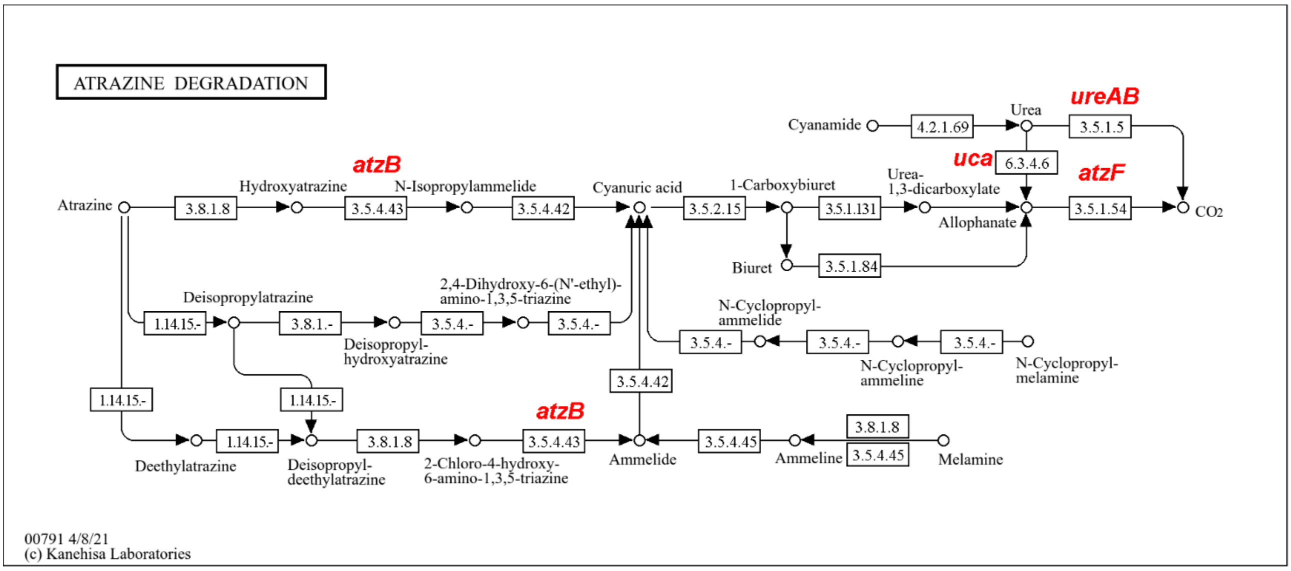

Figure S1 The atrazine degradation pathway map in KEGG pathway (map 00791).
